# Mangosteen ethanol extract alleviated the severity of collagen-induced arthritis in rats and produced synergistic effects with methotrexate 

**DOI:** 10.1080/13880209.2018.1506939

**Published:** 2018-12-04

**Authors:** Jian Zuo, Qin Yin, Lin Wang, Wen Zhang, Yan Fan, Yu-Yan Zhou, Yan Li, Guo-Dong Wang

**Affiliations:** a Yijishan Hospital of Wannan Medical College, Wuhu, China;; b The Second Affiliated Hospital of Wannan Medical College, Wuhu, China;; c Anhui Provincial Engineering Research Center for Polysaccharide Drugs, School of Pharmacy, Wannan Medical College, Wuhu, China

**Keywords:** Rheumatoid arthritis, α- mangostin, T cells, disease modifying antirheumatic drug

## Abstract

**Context:***Garcinia mangostana* Linn. (Guttiferae) pericarp is used as a traditional medicine in South Asia to treat inflammatory diseases.

**Objective:** This study investigates therapeutic effects of *G. mangostana* pericarp ethanol extract (MAN) on collagen-induced arthritis (CIA) and interactions with methotrexate *in vivo*.

**Materials and methods:** Male Sprague-Dawley rats with CIA were treated with MAN (0.5 g/kg/day), methotrexate (0.5 mg/kg, bw) or combination of both for 36 days, respectively (*n* = 8/group). Another eight healthy and CIA rats served as normal and model control, respectively. Therapeutic effects were evaluated based on paw edema and arthritis score during the experiment and serological markers at the end of the study period. Histological and radiological examinations were used to assess joint destructions. The immune status was investigated by immunohistochemistry and flow cytometry.

**Results:** All treatments decreased the arthritis score and paw inflammation in CIA rats. Combination regimen significantly reduced anti-cyclic citrullinated peptide antibody in CIA rats to 85.83% (*p* < 0.05) and notably alleviated synovial hyperplasia and cartilage degradation in joints. Different from methotrexate, MAN significantly augmented CD25^+^ cells distribution (from 2.72 to 3.35%) and IL-10 secretion (from 202.4 to 241.2 pg/mL) in CIA rat blood. Meanwhile, MAN induced a greater IL-17 decrease and a FOXP3 increase in immune organs than MTX. Reduced TLR4 and IL-17 expression and elevated FOXP3 expression in joints also occurred under MAN treatment.

**Conclusions:** MAN protected joints from destruction in CIA rats and exerted synergistic effects with methotrexate by improving immune microenvironment. The combination regimen could bring additional benefits to rheumatoid arthritis patients.

## Introduction

Rheumatoid arthritis (RA) is a common systemic autoimmune disease affecting 1% of the total population worldwide. It is typically characterized by chronic inflammation and deformation of joints and eventually leads to disability of limbs (Liu et al. 2013). The etiopathogenesis of RA is multifactorial and a result of complex interactions among genetic, environmental and stochastic factors. Under certain conditions, some infections trigger the dysregulation of the immune system and cause persistent inflammation and articular destruction (Mcinnes and Schett 2011). The most commonly used drugs in RA therapy are conventional synthesized disease-modifying antirheumatic drugs (cDMARDs). When disrupted at an early stage, cDMARDs treatments usually result in promising clinical and radiological outcomes (Machold 2010). The use of biologic DMARDs (bDMARDs) targeting defined pathogenic cytokines has flourished over recent years as a result of their theoretical advantages. However, several clinical studies showed no significant differences concerning therapeutic efficacies between the two types of medications in treating RA patients (Osiri et al. 2016). cDMARDs are inexpensive and efficacious, and therefore are commonly used as the first-line drugs in clinical practice until now. Optimization of these conventional regimens would be beneficial and reasonable at the present stage.

Previously, we demonstrated that xanthone derivatives had significant therapeutic effects on experimental arthritis *in vivo* (Zuo et al. 2014, 2015). α-Mangostin (MG), a naturally occurring prenylated xanthone, is deemed as an attractive experimental compound, owing to its high abundance and potential clinical efficacy. Our recent investigation (Zuo et al. 2018) found that different mechanisms were involved in the therapeutic actions of cDMARDs and MG on adjuvant-induced arthritis (AA) in rats. The typical cDMARD, leflunomide, potently inhibited the hyperactivated immune functions associated with AA and was effective against the secondary inflammation and polyarthritis. Conversely, MG efficiently alleviated acute inflammation, while had a milder influence on the immune system. Compared with leflunomide, MG treatment resulted in significant structural and functional improvements to joints (Zuo et al. 2018). Considering the notable protective effects on joints and different therapeutic mechanisms, the combination of MG and cDMARDs could possibly have synergistic effects.

As the main resource of MG, the inedible pericarp of mangosteen [*Garcinia mangostana* Linn. (Guttiferae)] has been used as a traditional medicine to cure inflammations, pains, wounds and infections for centuries in South Asia (Pedraza-Chaverri et al. 2008). Mangostin compounds are abundant in the extract. It is used in many countries as a functional food ingredient to promote health properties. Because of the potential antirheumatic properties of MG, MG-abundant mangosteen extract may also produce therapeutic effects for RA. Since the medicinal values of mangosteen extract are well known and it is easily accessible. Its clinical use is more practical than that of MG. Hence, the present study evaluates the effects of the ethanol extract of mangosteen (MAN) on collagen-induced arthritis (CIA) and investigates its possible synergistic effects with methotrexate (MTX, an anchor DMARD extensively used in clinical practice) *in vivo* in rats.

## Materials and methods

### Materials and reagents

Incomplete Freund's adjuvant (IFA) was purchased from Sigma-Aldrich (St. Louis, MO). Lyophilized immunization grade bovine type II collagen (CII) was obtained from Chondex (Redmond, WA). Antibodies used in the immunohistochemical assays were purchased from KeyGen Biotech (Nanjing, China).

### Preparation of the ethanol extract of mangosteen

Fresh mangosteens were bought from markets in Anhui Province in October 2017, and identified by Professor Jian-Wei Chen (College of Pharmacy, Nanjing University of Chinese Medicine, Nanjing, China). A voucher specimen of the samples (ID: MAN-2017-002) was deposited in the Herbarium Center, Wannan Medical College (Wuhu, China). The fruits were peeled and the obtained pericarp was air dried, milled and later soaked and percolated with 95% alcohol three times (10 × the sample weight each time). The filtrate was combined and evaporated by a rotation evaporator. Subsequently, the concentrated sticky extract was kept in a vacuum oven under 50 °C until achieving the constant weight. MAN (143 g) extracted from 1 kg of pericarp contained 11% MG based on high-performance liquid chromatography-ultraviolet detector (HPLC-UVD) analysis (Supplementary Figure S1) using a previously validated assay (Xu et al. 2017).

### Animals

Male Sprague-Dawley (SD) rats (180 ± 10 g, 6 weeks old) were purchased from Qinglongshan Experimental Animal Company (Nanjing, China). The animals were housed four rats per cage under strictly controlled environmental conditions (a 12 h light/dark cycle, 24 ± 2 °C and 50 ± 2% relative humidity), and fed with commercial pellet rodent food and water available *ad libitum*. The animals were acclimated for seven days prior to experimental procedures. All animal protocols were approved by the Ethical Committee of Yijishan Hospital, Wannan Medical College (No. YJS 2017-3-024) and were strictly in accordance with the Guide for the Care and Use of Laboratory Animals.

### Induction of collagen-induced arthritis and administration

The induction of CIA in rats was conducted according to the instructions of Chondex with minor modifications. Briefly, lyophilized CII was dissolved in 0.05 M acetic acid to produce a CII solution at the concentration of 2 mg/mL, after standing overnight under 4 °C. An equal volume of IFA was mixed into the CII solution and stirred continuously on ice with a Pellet Pestle motor (Kimble Chase, NJ) to obtain a milky homogeneous emulsion. The emulsion (0.1 mL) was injected into the back and base of the tail subcutaneously at multiple points with a Hamilton syringe. Seven days later, a booster injection was administered at the base of tail. Secondary inflammation developed in all animals approximately eight days after the first immunization.

The induced animals were randomly assigned into four groups (with eight rats each) and another eight normal rats were used as normal controls. Animals in the MTX group received MTX at the dose of 0.5 mg/kg twice per week (on Tuesday and Friday, respectively). MAN suspended in saline was given to the MAN group once a day at the dose of 0.5 g/kg. The combination group received the same treatments with MAN plus MTX, as depicted above. CIA models and normal controls were treated with 0.5% sodium carboxymethyl cellulose (CMC-Na) (1 mL/200 g) instead. All these treatments were administered intra-gastrically and lasted for 36 days.

### Basic assessment of experimental arthritis in rats

Following the induction of CIA, all animals were observed continuously to assess the development and progression of CIA. The severity of CIA was periodically evaluated based on paw swelling and arthritis score. Paw edema of rats was evaluated by measuring the volume of the right hind paw and a water displacement method was employed for this purpose. The arthritis score was assessed based on the independent observations of three scholars. Each paw was scored on a scale of 0–4, therefore meaning that the highest total score was 16. The assessment criteria were as follows: 0= no edema or any visual changes; 1= slight edema and limited erythema; 2= light edema and erythema; 3= obvious edema and significant erythema; and 4= severe edema and extensive erythema. To investigate the effects of treatments on radiological changes in the joints of CIA rats, lateral and frontal digital radiography (DR) examinations of the hind limbs were carried out one day ahead of sacrifice.

### Sacrifice and sampling

Following the completion of treatments, all rats were sacrificed under anesthesia with chloral hydrate (on the 37th day since the first treatment). Blood was taken *via* the abdominal aorta, and collected into tubes which are of either pro- or anti-coagulation, depending on the subsequent experiments. The coagulated blood was used to separate serum, which was then divided into aliquots and kept under −80 °C until further analyses. The uncoagulated blood was used for complete blood count (CBC) and T cell subset distribution analysis. The dead animals were promptly dissected. Main organs and hind paws were separated from the body, weighed immediately to calculate tissue indexes (tissue weight versus body weight), and then fixed with formalin for further histological and immunohistochemical examinations.

### Analyses of hematological parameters

One portion of whole blood was directly subjected to an automated hematology system (ADVIA 120, Bayer Diagnostics, Berlin, Germany) for CBC analysis. The results obtained were applied to analyze cell subset distribution in blood. Another portion was used for T cell subset distribution analysis with flow cytometry according to the manufacturer’s instructions (Multi-Sciences, Hangzhou, China). Briefly, whole leucocytes from the blood were obtained by centrifugation following the lysis of red blood cells. CD4 and CD25 antibodies (5 μL, tagged with phycoerythrin and allophycocyanine, respectively) were mixed into the re-suspended cells, and kept under room temperature in the dark for 30 min. Then, the stained leucocytes were subjected to flow cytometry (FACS Calibur system, Becton & Dickson, San Jose, CA) for quantitative analysis (Hu et al. 2014).

In this study, we used rheumatoid factor (RF), anti-cyclic citrullinated peptide antibody (aCCP) and interleukin-10 (IL-10) as biomarkers to indicate the severity of CIA and immune functions, and their levels in serum were determined with ELISA by using commercially available kits (Cusabio, Wuhan, China) strictly in accordance with the manufacturers’ instructions. The levels of aspartate transaminase (AST), alanine transaminase (ALT), creatinine (Cr) and blood urea nitrogen (BUN) in serum were determined using quantitative colorimetric assay kits supplied by the Jian Cheng Bioengineering Institute (Nanjing, China) to assess the possible damages to the liver and kidneys following treatments.

### Histological and immunohistochemical examinations

Histological examination was carried out to assess the pathological changes in joints and organs. The fixed specimens were embedded in paraffin (limbs were decalcified with EDTA for two weeks beforehand) and then cut into 5 μm sections and mounted on glass slides. Processed paraffin-embedded sections were stained with hematoxylin/eosin and observed using an Olympus BH-2 light microscope (Tokyo, Japan) for general evaluation. The evaluation of disease severity was performed in a blinded fashion by a scholar who had no information about treatment specifications based on three important parameters listed as follows: inflammatory cells infiltration, synovial hyperplasia and joint destruction.

Some other sections were deparaffinized and treated with 0.3% hydrogen peroxide in 60% methanol for 10 min under room temperature to deactivate the endogenous peroxidase. The specimens were then treated with citric acid (10 μM) for 1 h with microwave heating and subsequently incubated with normal goat serum, primary antibodies and then appropriate biotinylated secondary antibodies. After the peroxidase staining with diaminobenzidine, the signal of proteins was visualized by counterstaining with hematoxylin.

## Statistical analysis

Results were expressed as mean ± SD. Statistical differences among different groups were evaluated using one-way analysis of variance followed by the *post hoc* tests using the SPSS statistical analysis software (SPSS, Chicago, IL, version 14.0).

## Results

### The combination of MAN and MTX augmented the hepatotoxicity of MTX in rats

Our previous study showed that that MG at doses up to 50 mg/kg could cause liver damage (Zuo et al. 2018); however, we did not see such liver toxicity following treatments with MAN at the dose containing an equivalent amount of MG in the present study. This could be due to potential antagonisms among compounds in the extract. Meanwhile, we found that MAN induced heavy sweating in rats shortly after administration, presumably due to activation of the cholinergic neuronal system. MTX is known to cause damage to multiple organs; therefore, we optimized the treatment dose of MTX and found no obvious toxicity at the dose of 0.5 mg/kg twice a week. Despite both MAN and MTX were safe under the optimized doses, the combination of them caused an augmented toxicity. The repeated combination treatment induced ascites and decreased urine output, finally resulting in the death of rats (mortality: four of 10 rats during the treatment duration). Based on these observations, we assume that the detriments to the liver and kidneys likely account for the casualties. However, neither histological (Supplementary Figure S2) nor biochemical examinations (Table 1) showed any signs of renal injuries. But the heightened level of ALT in serum suggested that the combination treatment caused liver damage. Although the exact reason leading to increased hepatotoxicity of MTX was unknown, we hypothesized that it could be due to prolonged clearance of MTX under the combination treatment, as the side effects and death of rats usually occurred shortly after MTX administration and several previous studies have clearly shown significant pharmacokinetic interactions between polyphenols and MTX leading to a prolonged clearance of MTX (Chiang et al. 2005; Shia et al. 2013).

**Table 1.** Liver and renal functions of rats^a^.

	ALT (U/L)	AST (U/L)	BUN (mmol/L)	CR (μmol/L)
Normal	51.4 ± 5.2	100.6 ± 9.4	7.2 ± 0.2	24.9 ± 1.6
CIA model	44.5 ± 3.6	109.3 ± 12.6	8.0 ± 1.0	23.8 ± 1.4
MTX	55.8 ± 10.9	84.8 ± 8.8	6.8 ± 0.5	20.6 ± 2.8
MAN	59.5 ± 15.9	97.3 ± 25.3	7.7 ± 1.3	22.3 ± 1.8
MTX + MAN	94.3 ± 5.6***	116.3 ± 20.3	6.5 ± 0.8	20.9 ± 3.1

^a^Data are presented in mean ± SD.

*** *p* < 0.001 compared with CIA model.

### The combination of MAN and MTX improved therapeutic effects of MTX on CIA

CIA developed in all induced rats after the booster injection at day 4. CIA rats then suffered from severe inflammation afterwards. From day 23 onwards, the local inflammation of ankles was ameliorated, but significant bulbous swelling and deformation occurred in proximal interphalangeal joints. When at rest, the induced animals laid on one side due to pain. In addition, the pain appeared to make the rats limp and the motor function of limbs became limited.

**Figure 1.**
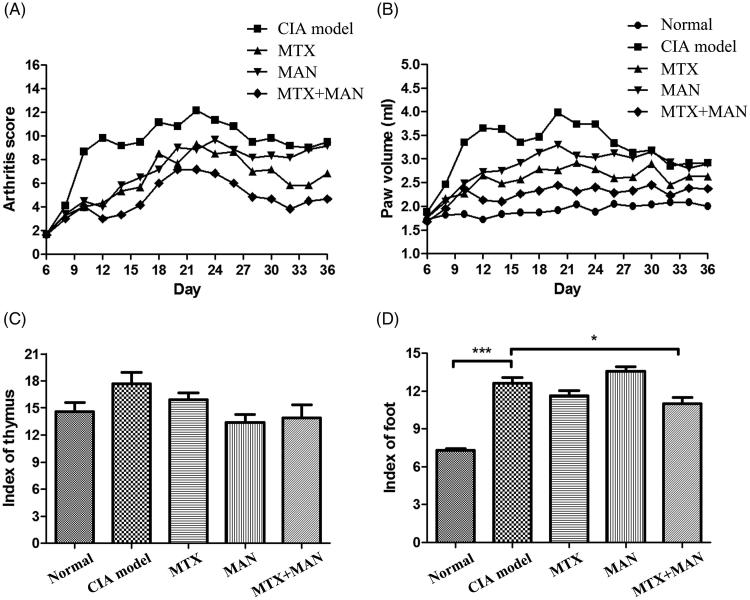
Overall severity of collagen-induced arthritis (CIA) in rats. (A) Arthritis score; (B) paw edema; (C) weight index of thymus and (D) weight index of hind foot. Statistical significance: *p < 0.05 and ***p < 0.001.

Similarly to MG, MAN was efficacious against inflammation especially at the early stage, indicated by the decreases of both arthritis score and paw volume. However, the efficacy was not maintained as CIA progressed. As a first-line DMARD, MTX performed well. The arthritis score of MTX-treated rats was significantly lower than the models throughout the observation period, as was the alleviation of paw swelling. The efficacy of MTX on CIA was further improved by the combination with MAN. The combination treatment suppressed the onset and progression of polyarthritis with the highest efficacy (Figure 1(A)). Compared with monotherapies, the combination therapy of MAN and MTX further reduced the local inflammation indicated by paw volume changes (Figure 1(B)). These results support the synergistic effects of MAN and MTX.

Tissue index results provided further evidence to support this conclusion. Hyperplasia of the thymus occurred following CIA induction and all the treatments restored the abnormal immunological changes (Figure 1(C)). Significantly increased paw weights indicated severe inflammation in CIA rats. The MTX treatment alone mildly reduced the severity of local inflammation, whereas MAN had no effect on paw edema. The combination regimen efficiently improved the therapeutic effects of MTX and resulted in a significant decrease of index of foot (Figure 1(D)).

### Effect of treatments on pathological changes of hematological parameters

CBC analysis demonstrated enlarged populations of white blood cells, lymphocytes, and monocytes in CIA rats. These hematological changes indicated inflammation and disruption of the immune balance *in vivo*, which was consistent with its clinical manifestation, further validating the successful development of CIA. All the treatments restored these pathological changes and the combination of MAN and MTX showed the most effective treatment outcomes (Table 2). These results provided direct evidence to support the synergy between MAN and MTX on CIA. Signs of anemia were observed in rats under the treatment with MTX, including the reduced levels of red blood cell and hemoglobin, confirming inhibition of MTX to hematopoiesis. MG ameliorated this effect under the combination treatment, exhibiting its possible antagonistic effect against the hematotoxicity of MTX.

**Table 2.** Results of CBC analysis^a^.

	WBC	NEU%	LYM#	MO#	RBC	HGB	MCH	PLT
Normal	12.9 ± 2.7	11.1 ± 3.8	10.4 ± 2.2	0.7 ± 0.3	7.7 ± 0.8	146.6 ± 11.8	19.1 ± 0.7	949.8 ± 173.7
CIA model	19.2 ± 4.5	16.8 ± 2.8	14.2 ± 3.9	1.6 ± 0.4*	7.1 ± 0.6	131.5 ± 7.9	18.6 ± 0.5	978.8 ± 221.8
MTX	15.7 ± 1.2	22.1 ± 7.3	10.8 ± 1.3	1.1 ± 0.2	6.9 ± 0.6	127.0 ± 11.9	18.5 ± 0.2	804.3 ± 311.1
MAN	17.0 ± 4.7	24.6 ± 10.8	12.0 ± 4.8	1.2 ± 0.4	7.6 ± 0.8	141.5 ± 16.3	18.7 ± 0.3	627.5 ± 255.9
MTX + MAN	14.6 ± 0.4	28.5 ± 13.4	9.5 ± 2.2	0.8 ± 0.2	8.1 ± 0.5	152.5 ± 9.3	18.8 ± 0.4	701.0 ± 335.0

^a^The unit was 10^9^/L. Data are presented in mean ± SD.

WBC: white blood cell; NEU%: percentage of neutrophil; LYM#: absolute lymphocyte counts; MO#: absolute monocyte counts; RBC: red blood cell; HGB: hemoglobin; MCH: mean corpuscular hemoglobin; PLT: blood platelet counts.

* *p* < 0.05 compared with normal control.

**Figure 2.**
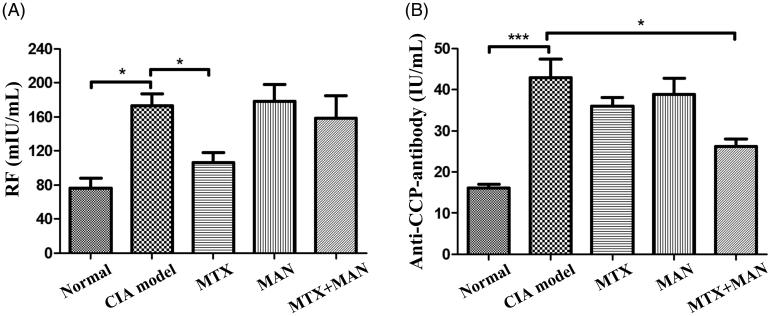
Levels of serological markers in serum of rats. (A) Level of rheumatoid factor (RF, mIU/mL)and (B) level of anti-cyclic citrullinated peptide antibody (anti-CCP-antibody, IU/mL). Statistical significance: *p < 0.05 and ***p < 0.001.

The evolution of RA is closely associated with autoantibodies which usually serve as diagnostic biomarkers for RA. RF and aCCP have been used widely in the clinical diagnosis of RA (Jaskowski et al. 2010). There is sufficient evidence, from both current and previous studies, supporting the relationship between the treatment with MTX and the decreased RF level (Alarcon et al. 1990; Świerkot et al. 2012). As xanthones possess significant pro-apoptotic effects on B cells (Azebaze et al. 2009), MAN is presumably able to suppress RF *in vivo;* however, we found in this study that MAN had no effect at all. This could be due to the limited exposure of circulatory B cells to free MAN. In the combination treatment, MAN even offset the effects of MTX on RF (Figure 2(A)). Until now, there have been no comprehensive investigation about the effects of xanthones on the secretion capability of B cells, hence we have no explanation regarding the exact mechanisms contributing to the antagonistic effect and further investigation is needed to solve the puzzle. Another significant improvement was achieved under the combination treatment, as it efficiently suppressed aCCP, while monotherapy with either MTX or MAN offered extremely weak effects (Figure 2(B)). Because of its high specificity and sensitivity in RA, the diagnostic use of aCCP is attractive. It is especially useful to be used as a serologic marker for the early diagnosis and prognostic prediction of joint destruction (Mimori 2005). However, the most available drugs, including some biologics, are not effective enough to suppress it (De Rycke et al. 2005). The improved effect on aCCP suggested that the combination treatment could possess potential advantages over the use of MTX alone for a better long-term prognosis. 

### Combination treatment resulted in superior protective effects on joint in CIA rats

**Figure 3.**
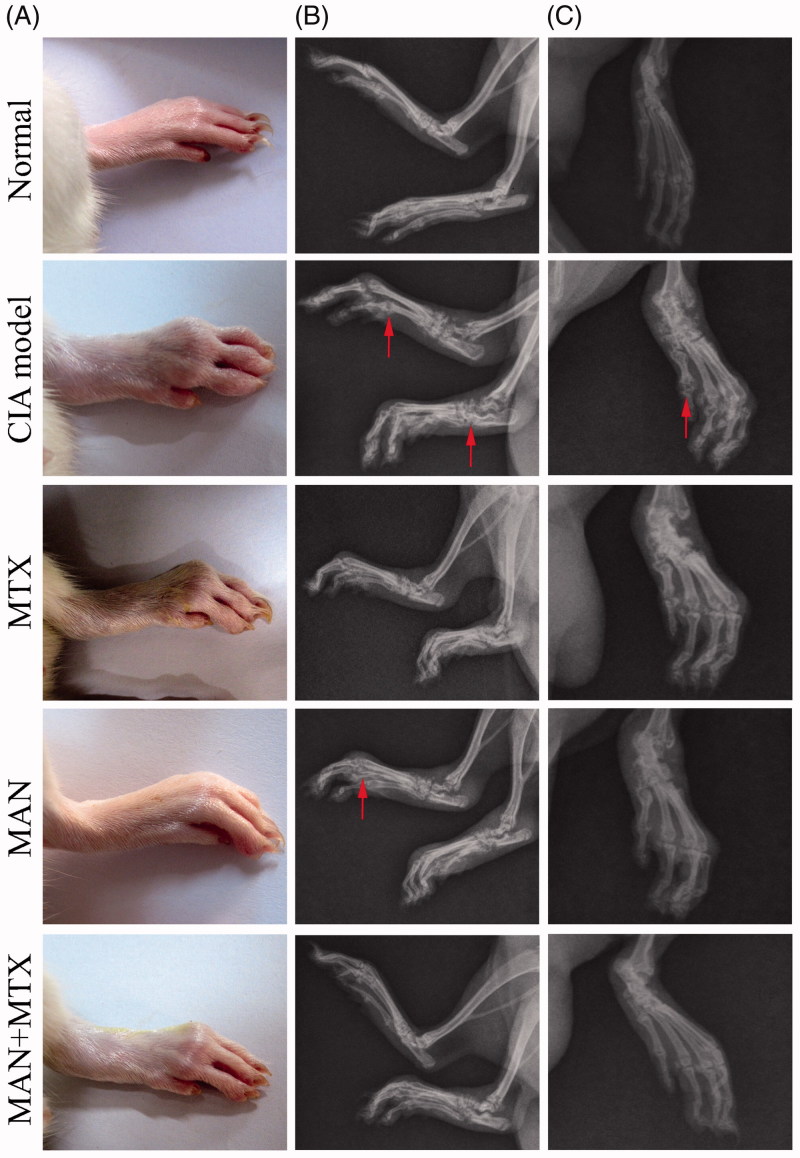
Effects of treatments on joint structure degradation in collagen-induced arthritis (CIA) rats. (A) Morphology examination of hind paw of rats; (B) lateral radiography image of hind limb of rats and (C) frontal radiography image of hind paw of rats. Red arrow: joint structure destruction.

As mentioned above, a significant improvement of clinical manifestations was achieved in CIA rats under MTX treatment, while the effects of MAN were less pronounced on polyarthritis and secondary inflammation. Meanwhile, we found that the deformation of joints in MAN-treated rats was greatly ameliorated when compared with other groups and the functional capacity of limbs was much better in the MAN group than the MTX-treated one. These facts suggest that, although the overall therapeutic efficacy of MAN was weaker than MTX, it could protect the joints with a high efficacy. Obvious synergistic effects of MAN and MTX were observed, as inflammation of joints was hardly observed in the combination treatment group by the end of the study (Figure 3(A)). To further assess the effects of treatments on joint destruction, we carried out DR examinations. This analysis revealed extensive damage to joints in CIA models, including soft tissue swelling, joint cavity narrowing, bone matrix resorption and osteophyte formation, which together led to a reduction in joint movement. All the changes were alleviated following the treatments, with MAN causing more prominent joint protection than MTX. Cartilage ossification and bone loss could still be found in the MTX group, especially at small joints, such as tarsal, metatarsal and interphalangeal regions, yet the conditions were much better under the MAN treatment. A superior radiological outcome was achieved following the combination treatment regimen (Figure 3(B,C)).

A previous study suggested that the RF titer would decide the radiographic damage in RA patients (Mewar et al. 2006); however, this study did not support any direct association between them. The RF level in CIA rats was raised significantly compared with the controls, whereas MTX deceased it a lot. DR examinations found no additional benefits brought by the significantly deceased RF under the MTX treatment when compared with the other groups. Meanwhile, we found that the combination of MTX and MAN notably suppressed the increased aCCP titer in CIA rats. It seems that this improved effect on aCCP could be associated with good radiological outcomes. Based on these results and literature data, we may conclude that bone erosion could be affected by multiple factors including RF, aCCP and other unknown factors. Together with some stochastic processes, this causes the inter-individual variations in the evolution of arthritis, leading to the unpredictability of arthritis prognoses with a small sample population. Even though the synergistic effects of MAN and MTX at the level of aCCP could benefit the radiological outcome in CIA rats, it was difficult to draw a solid conclusion based on the small sample size.

### MG treatment improved the pathological micro environment in joints of CIA rats

**Figure 4.**
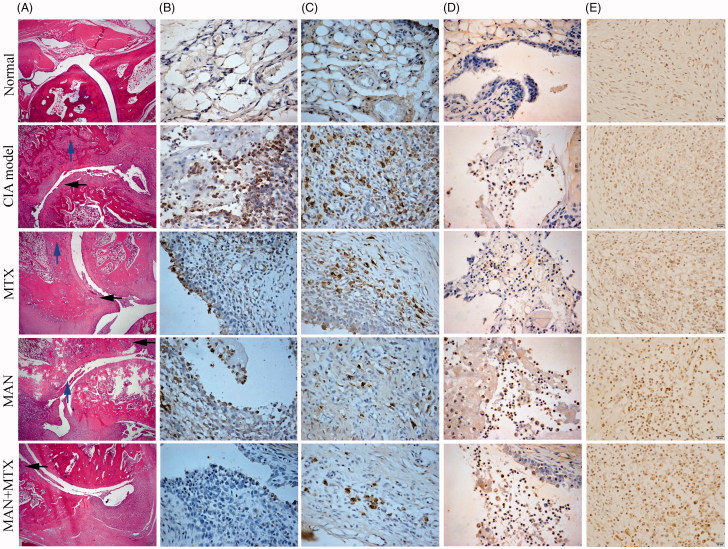
Effects of treatments on pathological changes of joints in rats. (A) Histological examination of ankle joints (black arrow: cartilage erosion and blue arrow: bone degradation); (B) expression of TLR4 in synovium; (C) production of IL-17 in synovium; (D) expression of FOPX3 in immune cells infiltrated into synovium and (E) expression of α7nAChR in synovium.

Histological examinations showed an intact joint structure in normal rats, while joints from CIA rats were severely damaged. Extensive infiltration of inflammatory cells was identified and the joint cavity was narrowed due to significant synovial hyperplasia. The erosive synovium invaded the cartilage and bone and caused degradation of joint structures. MTX and MAN both ameliorated the pathological conditions and the combination of both even augmented their effects (Figure 4(A)).

Our previous studies demonstrated that the protection of xanthones on joints resulted from their modulation of the NF-κB and MAPK pathways (Zuo et al. 2015; Ji et al. 2017; Zuo et al. 2018), both of which are downstream targets of TLR4 signaling. Consistent to RA, TLR4 was highly expressed in the synovium of CIA rats. MTX efficiently suppressed TLR4 expression and a similar effect of MAN was also observed. The combination regimen thoroughly abolished this signaling (Figure 4(B)). Because TLR4 is an important part of the innate immune system and accounts for the persistent activation of antigen presenting cells under RA conditions, abrogation of TLR4 signaling is essential to the alleviation of the disease *via* downregulation of some proinflammatory pathways and restoration of immune tolerance (Huang and Pope 2009). The synergistic effects of MAN and MTX on TLR4 promised better therapeutic effects *in vivo*.

IL-17 is a pathological cytokine mainly produced by Th17 cells, which is implicated in the initiation phase of RA. It not only perpetuates the chronic inflammation triggered by T cells, but also has a role in bone erosion, suggesting that anti-IL17 therapy is theoretically and practically effective (Lubberts et al. 2004). A high level of IL-17 was detected in the synovial membrane of CIA rats and all the treatments decreased the level of IL-17. Although MAN was unable to markedly decrease the number of lymphocytes, it suppressed the secretion of Th17 cells with a higher efficacy than MTX (Figure 4(C)). As IL-17 is situated at the top of the inflammatory cascade and manipulates the activation of many effector cells involved in bone degradation, inhibition of IL-17 is effective to ameliorate joint destruction (Kuligowska and Odrowaz-Sypniewska 2004). MAN induced a substantial decrease of the IL-17 level, thus protecting the joints.

Downregulation of TLR4 and IL-17 implied that MAN could improve the immune microenvironment in joints of CIA rats; however, the underlying mechanisms remain elusive. CD25^+^ Treg cells play a pivotal role in the control of the systemic symptoms in CIA by inhibiting the CII-specific antibody response. Meanwhile, they also affect the progression of bone erosion by controlling the recruitment and infiltration of inflammatory cells into the joints (Morgan et al. 2003). We found extensive T cell infiltration into the synovium in all animals except the normal controls. Immunohistochemical analysis illustrated that the expression of FOXP3 in joint cavity T cells was limited in the CIA and MTX groups, while its level was significantly increased following the treatment with MAN (Figure 4(D)). As MAN induced a significant activation of the cholinergic system, manipulation of the cholinergic anti-inflammatory pathway (CAP) could be implicated in these immune changes. Hence, we investigated the expression of the α7 subunit of the nicotinic acetylcholine receptor (α7nAChR) in synovium. As expected, MAN notably increased its expression in the immune cells that had infiltrated the synovium, while MTX had no effect (Figure 4(E)). Meanwhile, we noticed clear correlations among the expression of TLR4, IL-17, FXOP3 and α7nAChR. It seemed that the sustained activation of CAP was associated with the altered differentiation profile of T cells and favored for the improvement of local inflammatory conditions.

### MAN altered the profile of T cell differentiation in immune organs

**Figure 5.**
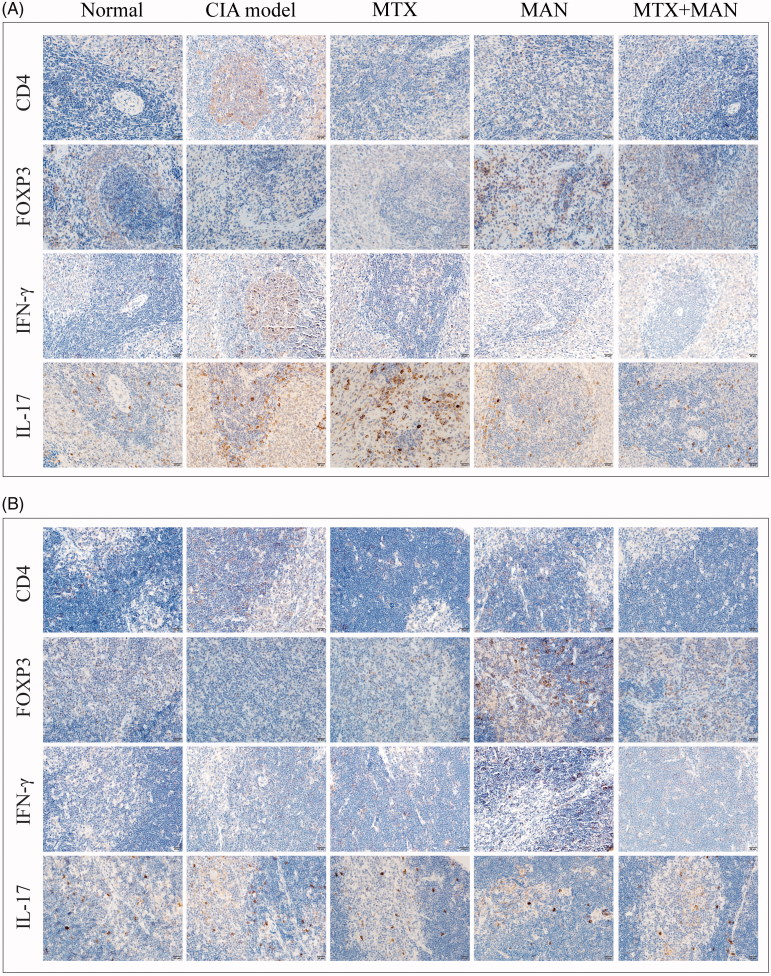
Effects of treatments on T-cell subsets in main immune organs (investigated by an immunohistochemical method). (A) Expression of CD4, FOXP3, IFN-γ and IL-17 in spleen and (B) Expression of CD4, FOXP3, IFN-γ and IL-17 in thymus.

We found that MAN greatly affected the immune milieu within joints. To further investigate its effects on immune functions, we examined the changes that occurred in immune organs. As no significant histological changes were observed (Supplementary Figure S2), we analyzed the distribution of T cell subsets in immune organs by immunohistochemical assays. Overall, all of the treatments significantly reduced the number of CD4^+^ cells in both the spleen and thymus of CIA rats; however, the treatments exhibited differential effects on different subsets. Th1 cells are important for the initiation of disruption to the immune system and the control of IFN-γ positive cells is critical for the alleviation of RA. Under the MAN and MTX treatments, IFN-γ positive cells were largely decreased in number and the combination treatment produced a greater effect. Consistent with the results from the immunohistochemical analysis of joints, the production of IL-17 was significantly heightened under CIA pathological conditions, which was then greatly reverted by MAN, while MTX exerted little effect. In addition, the results indicated an upregulated FOXP3 level by MAN in both the spleen and thymus, suggesting a functional recovery of Tregs from the depressed state in CIA rats (Figure 5). The simultaneously reduced secretion of IL-17 and IFN-γ and increased expression of FOXP3 implied that MAN could suppress pathogenic CD4^+^ cells, a feature in the development of RA *via* the upregulation of Tregs.

### MAN restored CD25^+^ cells in CIA rats

**Figure 6.**
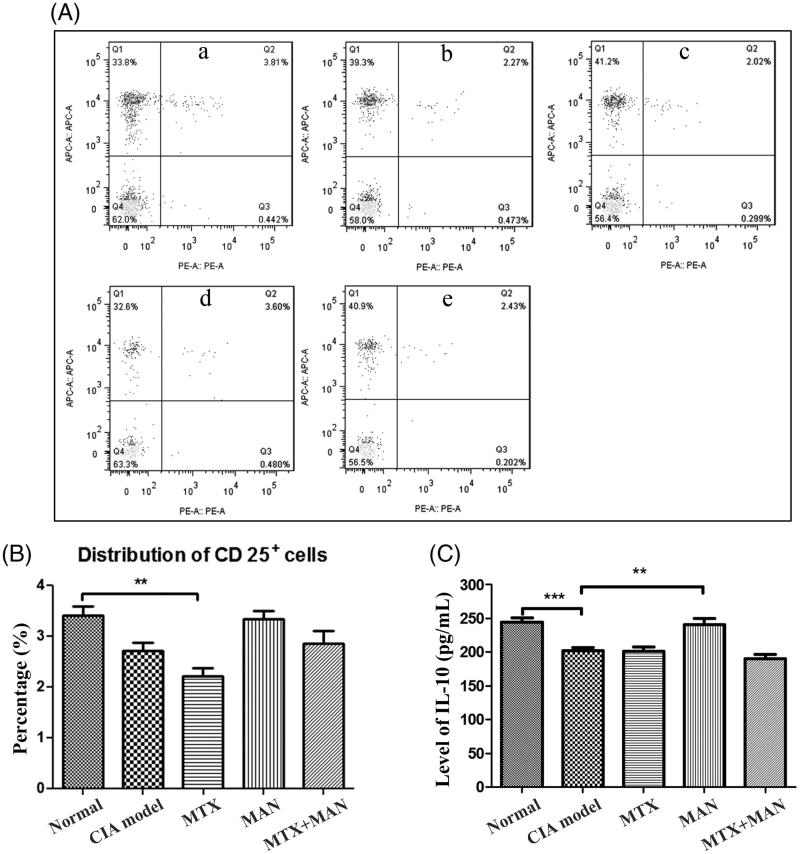
Effects of treatments on Treg cells in peripheral blood. (A) Distribution of CD25^+^ T cells subset in blood of rats; (B) quantification of flow cytometric assay and (C) level of IL-10 in serum of blood (determined by the ELISA method). Statistical significance: **p < 0.01 and ***p < 0.001.

As we depicted above, MAN may restore immune tolerance *via* modulating the Th/Treg cell ratio and the upregulation of Tregs was important to the restoration of immune homeostasis *in vivo*. To further validate this assumption, we investigated the distribution of CD25^+^ cells in the peripheral blood. The subset of CD25^+^ Tregs was reduced in CIA rats and MTX even further strengthened the tendency. MAN raised the level of CD25^+^ Treg cells to near that of the untreated group (Figure 6(A,B)). As the functions of Tregs are compromised under some pathological conditions, we investigated the level of IL-10 in serum, which is known to have a suppressive role in the etiology of RA and is primarily produced by Tregs. Consistent to its effects on Tregs distribution, MAN significantly induced the IL-10 level, indicating a functional restoration of Treg cells (Figure 6(C)). These lines of evidence, together with the findings mentioned above, demonstrated that MAN induced a substantial population increase and functional recovery of Tregs, which then contributed to the improvements of immune-mediated systemic symptoms and joint destruction in CIA rats.

## Discussion

As a versatile bioactive naturally occurring compound, MG has a great potential in the therapy of RA. However, according to its chemical structure, MG is a strong lipophilic agent with poor water-solubility and possesses significant pharmacokinetic disadvantages, such as poor gastrointestinal absorption and short half-life (Li et al. 2011). Due to the sophisticated solubilization effects that occur in the extract, it would be helpful to use MAN instead, as MAN is more easily dispersed in water and administered. Since oral administration of the extract would increase the free compound exposure, MAN appeared to be better than pure MG (Li et al. 2013). Hence, we examined the therapeutic effects of MAN in this study. We realized that, although MG containing product was an efficient anti-inflammatory agent, its use as a single agent had substantial shortcomings, suggested by its weak effects on secondary polyarthritis in experimental arthritis models. The efficacy of DMARDs is well recognized by rheumatologists. These drugs are commonly used in clinical practice and the combination with other antirheumatic drugs usually offers better clinical outcomes. Herein, we performed this study to optimize the most commonly used regimen based on the combination of MTX and MAN.

The results obtained strongly support the synergistic effects of MTX and MAN on CIA in rats; however, MAN also increased the toxicity of MTX. We assumed that these effects were at least partly due to the changes in pharmacokinetics of MTX caused by MAN. Two groups of SD rats were used to test this assumption. Some rats were pretreated with MAN (0.5 g/kg once a day) for seven days. Then, all the animals were fasted overnight and received one single oral dose of MTX (2 mg/kg). Blood was collected serially at pre-arranged time intervals and HPLC was employed to monitor the concentrations of MG in the plasma. We found that pretreatment with MAN obviously elevated the levels of unconjugated MTX in the plasma, and increased the AUC approximately by four-fold (Supplementary Figure S3). This suggested that MAN can significantly alter the pharmacokinetics of MTX, leading to the improvement of therapeutic efficacy *in vivo*, but also increase the risk of toxicity. Therefore, the therapeutic dose of MTX should be further investigated until an optimal dose is identified, for use in combination treatment, in order to achieve an optimal therapeutic efficacy and avoid potential toxicity.

Although an increasing amount of evidence suggest that anti-inflammatory effects could be the major mediators of its actions in RA (Brown et al. 2016), modulation of immune functions by MTX is still advantageous in controlling autoimmune-mediated joint degradation. Because of their notable established role in the pathogenesis of RA, Th1 cells are deemed as an important therapeutic target (Herman et al. 2008). However, recent studies have indicated that RA is more than likely a Th17 cell-mediated disease. Th17 cells cooperate with synovial fibroblasts in a pro-inflammatory feedback loop, perpetuating chronic inflammation and driving RA progression (van Hamburg et al. 2011). Treatment with MTX reduced the production of both IFN-γ and IL-17, but this also has a negative effect on FOXP3^+^ cells. It suggested that MTX could be an immunosuppressant specifically targeting CD4^+^ T cells. Compared with MTX, the effects of MAN on T cells were selective. Likewise, MAN also negatively regulated Th1 and Th17 cells, but meanwhile it promoted the differentiation of CD25^+^ FOXP3^+^ cells. The population and function restoration of Tregs possessed an important complementary immunoregulatory effect with MTX. In this study, we showed evidence that MAN was a specific inducer of immunotolerance. The combined use of MAN with MTX could strengthen the suppressive effects of MTX on Th1/17 cells and offer an additional synergistic therapeutic effect by promoting the differentiation and functions of Tregs.

Notable improvements were brought about by the MAN treatment concerning the rapid anti-inflammation and joint protection effects. Inspired by the heavy sweating induced by MAN, we thought these effects could be associated with the manipulation of the CAP pathway, in which the biological function is based on the interactions between acetylcholine and α7nAChR found on the surface of both fibroblast-like synoviocytes and immune cells. In recent years, CAP has been suggested as a promising therapeutic target in treatments of RA (van Maanen et al. 2009). We found that MG significantly upregulated the expression of α7nAChR in the synovium and synchronously improved the local immune milieu. These changes subsequently contributed to the alleviation of inflammation and tissue destruction in joints. Changes that occurred in the expression of TLR4 provided us with additional evidence to support this notion. As it is believed that RA is initiated by some unknown infections, TLR4 could perpetuate the immune disruption *in vivo* in the initial stage of the disease. As an important component of the innate immune system, TLR4 plays an important role in acute inflammatory responses and infectious defense. Meanwhile, TLR4 is also implicated in some chronic immune diseases *via* balancing T cell subsets (Abdollahi-Roodsaz et al. 2008). Activation of α7nAChR could inhibit TLR4 expression and its downstream pathways (Kim et al. 2014). Therefore, we hypothesize that that the activation of CAP by MAN should account for the inhibition of TLR4 at least partially, which subsequently restores the immune homeostasis. Future studies should be done to further validate this hypothesis.

## Conclusions

The current study revealed the substantial therapeutic effects of MAN on CIA in rats with a great potential for clinical applications in the therapy of RA. MAN was especially efficient against the acute inflammation and joint destruction associated with RA, which was possibly due to the improvement of the immune microenvironment *via* activation of the CAP pathway. Because of the significant complementary activity between MAN and MTX in treatments of CIA, the combined use of them would offer synergistic effects *in vivo*.

## Supplementary Material

Supplementary Figures S1-S3
