# Supplementary material for: Mangosteen ethanol extract alleviated the severity of collagen-induced arthritis in rats and produced synergistic effects with methotrexate
Source: Pharm Biol. 2018 Dec 4;56(1):455–64. doi: 10.1080/13880209.2018.1506939 (PMC6282431; doi:10.1080/13880209.2018.1506939)
Supplement: Supplementary Figures S1-S3 [file IPHB_A_1506939_SM9194.docx]

**Figure S1.** HPLC-UVD Chromatograms of both MAN and MG


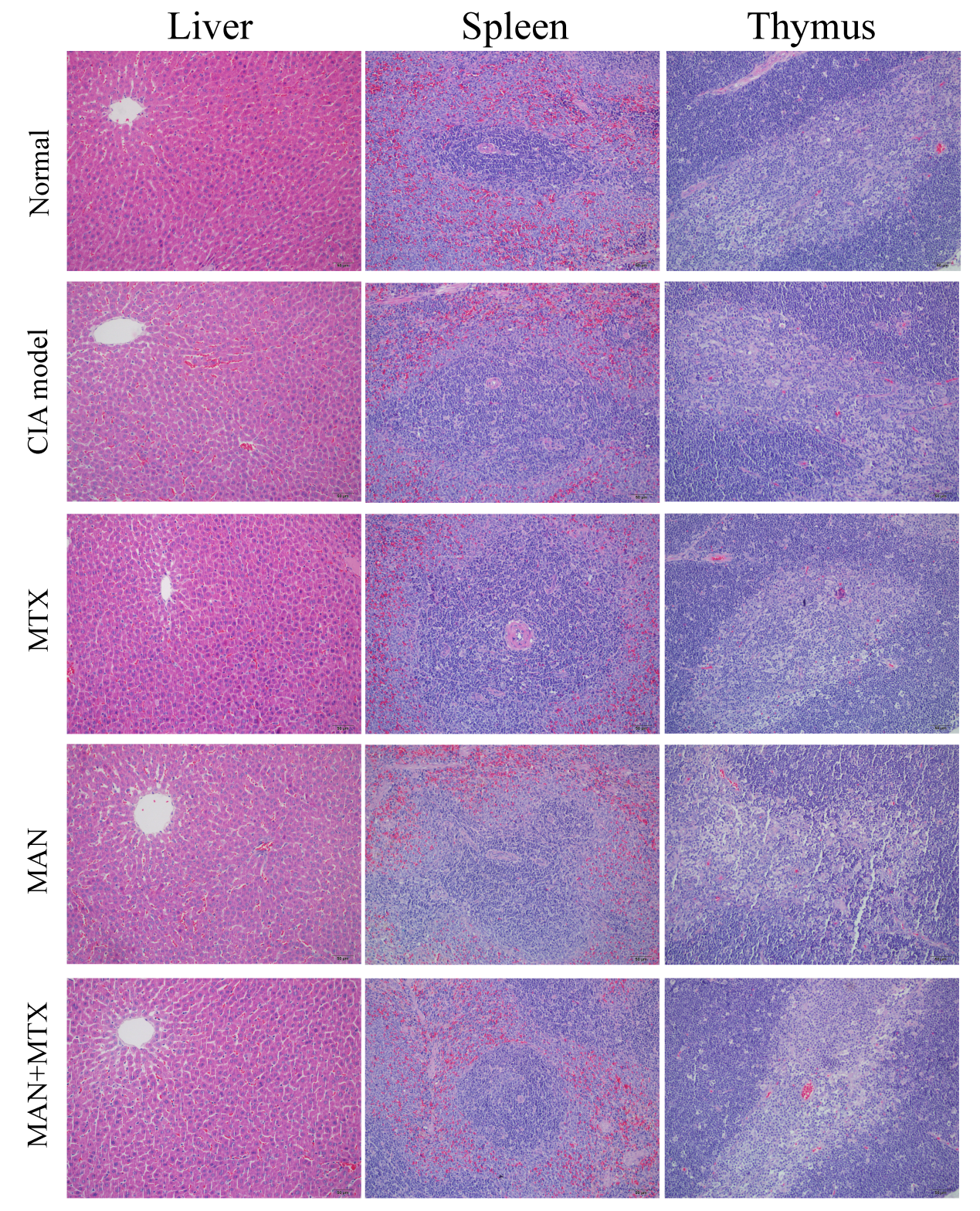


**Figure S2.** Histological examination of main organs in rats.


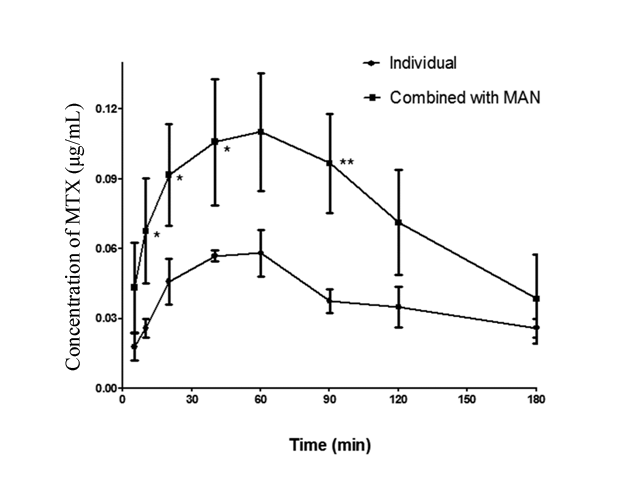


**Figure S3.** Mean plasma concentration of methotrexate (MTX) in rats versus time after one single oral dose (1 mg/kg) with/without the combination with MAN. Statistical significance: **p < 0.05* and *** p < 0.01*.
